# Application of the ISBAR bedside handover communication model in a pediatric hematology–oncology department: a pre–post intervention study

**DOI:** 10.3389/fped.2026.1785680

**Published:** 2026-03-06

**Authors:** Junxia Zhou, Jingjing Ma, Huaying Li, Long Li

**Affiliations:** 1 Department of Pediatric Hematology and Oncology, West China Second University Hospital, Sichuan University, Chengdu, Sichuan, China; 2 Key Laboratory of Birth Defects and Related Diseases of Women and Children, Ministry of Education, Sichuan University, Chengdu, Sichuan, China

**Keywords:** bedside handover, communication, hematology–oncology, ISBAR, pediatric nursing

## Abstract

**Background:**

Pediatric patients in hematology–oncology departments often present with complex, severe, and rapidly changing conditions, which place high demands on the accuracy and completeness of nursing handovers. Traditional bedside handovers are prone to information omission and unclear prioritization, potentially compromising patient safety. The ISBAR (Identification–Situation–Background– Assessment–Recommendation) communication model is a structured framework designed to standardize clinical handovers.

**Objective:**

To evaluate the effects of implementing the ISBAR bedside handover communication model on handover quality in a pediatric hematology–oncology department.

**Methods:**

A pre–post intervention study was conducted among nurses in the pediatric hematology–oncology department of a tertiary hospital in Sichuan Province, China. The ISBAR communication model was introduced into routine bedside handovers. Nurses’ bedside handover satisfaction, critical thinking disposition, and incidence of nursing handover problems, as well as patients’ family members’ perceptions of bedside handover, were assessed before implementation and three months after implementation.

**Results:**

After implementation of the ISBAR model, nurses’ bedside handover satisfaction scores increased significantly compared with baseline (75.20 ± 4.84 vs. 68.82 ± 7.43, *P* < 0.05). Nurses’ critical thinking disposition scores also improved significantly (269.28 ± 31.05 vs. 248.10 ± 42.63, *P* < 0.05). Family members’ perception scores of bedside handovers increased markedly after the intervention (78.69 ± 8.09 vs. 54.43 ± 18.79, *P* < 0.05). The incidence of nursing handover problems decreased from 21.57% before implementation to 13.24% after implementation (*P* < 0.05).

**Conclusion:**

Implementation of the ISBAR bedside handover communication model in a pediatric hematology–oncology department significantly improved handover quality, enhanced nurses’ critical thinking disposition, increased satisfaction among nurses and patients’ family members, and reduced nursing handover problems.

## Introduction

Nursing handover is a fundamental and critical routine practice in clinical nursing, playing an essential role in ensuring the continuity and integrity of patient care plans. A standardized and effective handover ensures the accurate transfer of critical patient information across different nursing stages, facilitating the continuous and coherent execution of nursing and therapeutic activities (1). A clear and explicit communication model during handover provides both parties involved in the transfer of nursing responsibility with ample opportunities to clarify information, ensuring a unified and comprehensive understanding of the patient's condition and key nursing points (2, 3). Inaccurate or incomplete information transfer during the handover process can lead to gaps in patient information, affecting the scientific and accurate nature of subsequent nursing decisions, which in turn threatens patient safety, disrupts the normal diagnostic and treatment processes, and may even cause severe adverse consequences (4, 5). In nursing practice, even minor oversights or errors can have irreversible effects on patients (6, 7). As a key component of nursing work, the quality of handovers directly impacts the overall quality of nursing care and patient satisfaction (8).

The ISBAR (Identification, Situation, Background, Assessment, and Recommendation) standardized communication model is composed of five key elements: Identification (I), Situation (S), Background (B), Assessment (A), and Recommendation (R) (9). Originating from the U.S. Navy submarine forces, the ISBAR model is centered around a structured framework that standardizes the information transfer process, ensuring the integrity and consistency of the handover content, thus reducing misunderstandings and errors, and improving communication efficiency within medical teams and the quality of nursing care (10, 11). Research indicates that the ISBAR model helps reduce medical errors resulting from communication breakdowns, enhances team collaboration, and improves patient treatment outcomes and safety (11, 12). Previous studies have shown that the ISBAR communication model has been successfully applied in various clinical departments, such as the ICU (13), rheumatology and immunology departments (14), and operating rooms (11), demonstrating its effectiveness in improving the quality of nursing handovers (15).

Therefore, this study aims to explore the application of the ISBAR bedside handover communication model in the pediatric hematology-oncology department to provide evidence for optimizing nursing handover processes, improving nursing quality, and ensuring patient safety. While ISBAR is widely utilized globally, its application within the specific cultural and clinical context of pediatric hematology-oncology in West China remains underexplored. This specialty is characterized by long-term treatments, complex chemotherapy protocols, and high family involvement, creating unique communication challenges distinct from general adult wards. This study bridges this gap by evaluating the adaptability and impact of ISBAR in this high-stakes setting.

## Methods

### Study design

A pre–post intervention study design was adopted.

### Participants

This study was approved by the Ethics Committee of a tertiary hospital in Sichuan Province, China (Approval No. Medical Research 2024 Round Approval No.094). Nurses working in the pediatric hematology–oncology department between May and August 2023 were recruited.

Inclusion criteria were as follows:

1. possession of a valid nursing license;
2. at least 6 months of continuous work experience in the pediatric hematology– oncology department;
3. ability to participate in bedside handovers;
4. voluntary participation with informed consent.

Exclusion criteria were:

1. inability to participate throughout the study period due to training, rotation, or long-term leave;
2. withdrawal from the study during the intervention period.

A census sampling method was employed to recruit participants. The total number of registered nurses in the pediatric hematology-oncology department during the study period was 60. All 60 nurses met the inclusion criteria, were approached, and voluntarily agreed to participate. No participants withdrew from the study or were lost to follow-up. Thus, 60 nurses completed both the pre- and post-intervention assessments, yielding a response rate of 100%. Among them, 59 were female (98.33%) and 1 was male (1.67%). The mean age was 29.28 ± 5.69 years (range: 23–43 years). Educational background included postgraduate degree (3.34%), bachelor's degree (73.33%), and junior college degree (23.33%). Nursing levels ranged from CN0 to CN3, and professional titles included nurse, senior nurse, and supervisor nurse.

### Pre-intervention handover practice

Prior to the implementation of ISBAR (May 2023), the routine bedside handover was conducted using a traditional, unstructured approach. The outgoing nurse would verbally narrate the patient's condition based on memory and personal notes, without a standardized sequence. This method often lacked specific headings for “Background” or “Recommendation,” “leading to variability in the information transferred.”

### Development of the ISBAR bedside handover process

Based on routine bedside handover practices, the ISBAR standardized communication model was introduced. During morning bedside handovers, outgoing nurses reported patients' conditions according to the ISBAR structure:

Identification: patient name, sex, age, bed number, and hospital identification number;

Situation: current condition, vital signs (temperature, heart rate, respiratory rate, blood pressure, oxygen saturation), pain status, and presence of bleeding or infection;

Background: medical history, primary diagnosis, treatment course, allergy history, and key psychological concerns;

Assessment: comprehensive evaluation of the patient's overall status, including level of consciousness, skin condition, catheter status, and potential nursing risks;

Recommendation: nursing priorities, precautions, potential adverse events (e.g., chemotherapy-related adverse reactions, transfusion reactions), and corresponding preventive measures.

### Training on the ISBAR bedside handover model

Before implementation, all participating nurses received standardized training. A training team led by the head nurse was established to organize and supervise the training process. Training content included the concept, significance, structure, and application of the ISBAR model in pediatric hematology–oncology bedside handovers.

Training methods included lectures, case analysis, scenario simulation, and video-based learning. After training, nurses were assessed through oral questioning and on-site simulations. Each nurse was required to complete at least five standardized ISBAR bedside handovers before being allowed to enter the formal implementation phase.

### Implementation of the ISBAR bedside handover

The ISBAR bedside handover model was formally implemented in May 2023. During daily morning handovers, outgoing nurses conducted bedside handovers using the ISBAR structure. Incoming nurses were encouraged to ask questions to clarify unclear information, ensuring completeness and accuracy of handover content. The intervention lasted for three months.

## Outcome measures

### Incidence of nursing handover problems

A self-designed checklist for bedside handover in pediatric hematology– oncology was used. Handover problems included omissions of important information and failure to communicate changes in treatment or nursing measures. Each identified problem was recorded as one case. The incidence rate was calculated as: number of handover problems/total observed handovers×100%.

### Nurses' critical thinking disposition

Critical thinking disposition was assessed using the Chinese Version of the Critical Thinking Disposition Inventory (CTDI-CV) (16), which includes seven dimensions and 70 items. Each item is rated on a 6-point Likert scale, with total scores ranging from 70 to 420. Higher scores indicate stronger critical thinking disposition.

### Nurses' bedside handover satisfaction

Nurses' satisfaction was evaluated using the Chinese version of the Nurse Assessment of Shift Report (NASR) (17), consisting of 17 items across five dimensions. Items are rated on a 5-point Likert scale, with higher scores indicating greater satisfaction.

### Family members’ perceptions of bedside handover

Family members' perceptions were measured using the Chinese version of the Patients' Views of Nursing Bedside Report (PVNC-BR) (18), which includes 17 items across three dimensions. Higher scores indicate more positive perceptions.

### Data collection and statistical analysis

Data were collected before implementation and three months after implementation. To minimize social desirability bias and ensure confidentiality, all questionnaires (CTDI-CV, NASR, PVNC-BR) were distributed and collected anonymously. Participants were instructed not to include any personal identifiers on the survey forms. The completed questionnaires were placed in a sealed box designated for research data to ensure that responses could not be traced back to individual nurses. SPSS 25.0 was used for statistical analysis. Categorical variables were expressed as frequencies and percentages and compared using the chi-square test. Continuous variables were expressed as mean ± standard deviation and compared using paired t tests. A two-sided *P* value < 0.05 was considered statistically significant.

## Results

### Comparison of the incidence of nursing handover issues

The incidence of nursing handover issues was compared before and after the implementation of the ISBAR bedside handover model. The results showed that the incidence of nursing handover issues was 21.57% before the intervention and 13.24% after the intervention. The incidence significantly decreased after the intervention, with a statistically significant difference (*P* < 0.01), as shown in Table 1.

**Table 1:** Comparison of the incidence of bedside handover issues between two groups (*n*, %).

Group	Number of handover observations	Number of issues	*χ^2^*	*P*
Pre-intervention	102	44 (21.57%)	6.243	0.012
Post-intervention	102	27 (13.24%)		

### Comparison of nurses' critical thinking ability

The Chinese version of the Critical Thinking Disposition Inventory (CTDI-CV) was used to assess the critical thinking ability of the same group of nurses both before and 3 months after the intervention. The results showed that the total score for nurses' critical thinking ability was 269.28 ± 31.05 after the intervention, which was significantly higher than the pre-intervention score of 248.10 ± 42.63, with a statistically significant difference (*P* < 0.05). In the six dimensions—seeking truth, open-mindedness, analytical ability, self-confidence, curiosity, and cognitive maturity—the post-intervention scores were all higher than the pre-intervention scores, with statistically significant differences (*P* < 0.05). In the dimension of systematization, the post-intervention score was higher than the pre-intervention score, but the difference was not statistically significant (*P* > 0.05), as shown in Table 2.

**Table 2:** Comparison of critical thinking scores between Two groups (score, mean ± SD).

Item	Pre-intervention	Post-intervention	*t*	*P*
Total CTDI-CV score	248.10 ± 42.63	269.28 ± 31.05	−3.224	0.002
Seeking truth	31.95 ± 8.04	34.80 ± 5.78	−2.131	0.037
Open-mindedness	29.88 ± 8.27	34.98 ± 8.02	−3.522	0.001
Analytical ability	33.97 ± 7.20	37.55 ± 4.98	−3.343	0.001
Systematization	35.25 ± 6.36	37.28 ± 5.22	−1.909	0.061
Self-confidence	40.33 ± 7.70	43.62 ± 6.68	−2.733	0.008
Curiosity	36.90 ± 6.37	38.80 ± 4.85	−2.051	0.045
Cognitive maturity	39.82 ± 6.43	42.25 ± 3.93	−2.539	0.014

### Comparison of nurses' bedside handover satisfaction

The Nurse Assessment of Shift Report (NASR) was used to assess the satisfaction of the same group of nurses with bedside handover before and 3 months after the intervention. The results showed that the total score for nurses' satisfaction with bedside handover was 75.20 ± 4.84 after the intervention, which was significantly higher than the pre-intervention score of 68.82 ± 7.43, with a statistically significant difference (*P* < 0.05). In the four dimensions—effectiveness and efficiency of the handover, promoting patient participation, increasing nurse responsibility for supervision and collaboration, and providing necessary information for the patient—the post-intervention scores were all higher than the pre-intervention scores, with statistically significant differences (*P* < 0.05). In the dimension of “ensuring patient safety,” the post-intervention score was higher, but the difference compared to pre-intervention was not statistically significant (*P* > 0.05), as shown in Table 3.

**Table 3:** Comparison of nurses’ bedside handover satisfaction between two groups (score, mean ± SD).

Item	Pre-intervention	Post-intervention	*t*	*P*
NASR total score	68.82 ± 7.43	75.20 ± 4.84	−5.537	<0.001
Effectiveness and efficiency of handover	8.60 ± 1.11	9.58 ± 0.79	−5.456	<0.001
Ensuring patient safety	8.85 ± 1.04	9.20 ± 0.97	−1.775	0.081
Promoting patient participation	8.38 ± 1.11	9.30 ± 0.94	−5.333	<0.001
Increasing nurse responsibility for supervision and collaboration	21.33 ± 2.58	23.40 ± 2.10	−4.682	<0.001
Providing necessary information for the patient	21.65 ± 2.59	23.72 ± 1.68	−5.363	<0.001

### Comparison of patients' family members' perception of bedside handover

The Patients' Views of Nursing Bedside Report (PVNC-BR) was used to assess the perception of bedside handover by the family members of the pediatric patients before and 3 months after the intervention. A total of 105 questionnaires were distributed before the intervention, with 100 valid responses returned; 105 questionnaires were distributed after the intervention, with 98 valid responses returned. The results showed that the total score for the family members' perception of bedside handover was 78.69 ± 8.09 after the intervention, which was significantly higher than the pre-intervention score of 54.43 ± 18.79, with a statistically significant difference (*P* < 0.05). In the three dimensions—interactive handover, respect and listening, and coordination of handover—the post-intervention scores were all significantly higher than the pre-intervention scores, with statistically significant differences (*P* < 0.05), as shown in Table 4.

**Table 4:** Comparison of patients’ family members’ perception of bedside handover between two groups (score, mean ± SD).

Item	Pre-intervention	Post-intervention	*t*	*P*
PVNC-BR total score	54.43 ± 18.79	78.69 ± 8.09	−12.28	<0.001
Interactive handover	19.41 ± 6.69	27.40 ± 3.29	−11.18	<0.001
Respect and Listening	12.73 ± 4.56	18.61 ± 2.02	−12.20	<0.001
Coordination of handover	22.28 ± 8.12	32.68 ± 3.23	−12.02	<0.001

## Discussion

The incidence of nursing handover issues significantly decreased after the implementation of the ISBAR bedside handover model. The ISBAR bedside handover helps avoid the omission of critical information due to unclear communication, making the key points of the handover more prominent and the nursing recommendations more targeted, thereby improving the quality of handover and ensuring medical and nursing safety (19). The structured handover using ISBAR reduces interference during the handover process, allowing both parties to more clearly identify the critical information to be verified, thereby lowering the risk of handover errors (11, 19). As a standardized communication tool, ISBAR not only improves handover quality but also enhances patient safety and reduces the occurrence of nursing adverse events (11, 19).

After the implementation of the ISBAR bedside handover model, nurses' critical thinking ability showed a certain degree of improvement, but overall, the scores remained at a moderate level before and after the intervention. Previous studies have indicated that critical thinking ability among nurses in internal medicine is relatively insufficient (20), which aligns with the characteristics of the department in this study.

Through the ISBAR bedside communication model, nurses are required to systematically organize and comprehensively assess the patient's condition during handovers. Their understanding of the nursing plan and their ability to receive the latest information directly affect their perception of handover quality (21). The structured handover process encourages nurses to be more proactive when analyzing the condition, identifying potential risks, and providing nursing recommendations, which helps improve their confidence, cognitive maturity, curiosity, analytical ability, and other dimensions of critical thinking. However, the improvement in critical thinking ability is long-term and systematic; relying solely on one communication model is unlikely to result in significant improvement. Continued efforts, such as evidence-based nursing training and scenario-based simulation teaching, are needed to sustain progress.

Nurses' satisfaction with bedside handover significantly increased after the implementation of the ISBAR model, which is consistent with previous studies (22–24). Some studies have indicated that after the introduction of ISBAR, most healthcare professionals believed that the overall quality of handover communication improved (9). The ISBAR handover model provides nurses with a unified and clear structural framework for handovers, making the handover content more organized and standardized. This helps nurses quickly and accurately communicate patient conditions, treatment progress, and potential risks, reducing information omissions and repetitions, and improving handover efficiency. The structured handover also helps the receiving nurse quickly understand the patient's condition and make accurate judgments, while establishing a mutual supervision mechanism during the handover process, thus enhancing nursing safety. The improvement in nurses' sense of control over handover quality and patient safety contributes to increased job satisfaction and a sense of professional achievement. In this study, improvements in the dimensions of handover effectiveness and efficiency, ensuring patient safety, promoting patient participation, increasing nurses' responsibility for supervision and collaboration, and providing necessary information for patients all reflect the positive impact of the ISBAR model on bedside handover quality in multiple areas (22–24).

After the implementation of the ISBAR bedside handover communication model, the perception of bedside handover by patients' family members significantly improved. Previous studies have shown that ISBAR bedside handover helps family members clearly understand the child's condition, treatment plan, and key nursing points, providing them with necessary guidance and reducing concerns, thereby enhancing overall satisfaction (25). The ISBAR model emphasizes structured and interactive handovers at the child's bedside, allowing family members to directly participate in the handover process, receive key information in a timely manner, and ask questions. This helps alleviate anxiety and improve cooperation. In the pediatric hematology-oncology department, where nursing dependency is high, it is particularly important for family members to understand infection prevention, bleeding precautions, and specialized nursing knowledge. The ISBAR bedside handover provides an effective way for family members to systematically understand key nursing points, thus enhancing their perception of the bedside handover.

### Limitations

This study has several limitations. First, it was conducted in a single center with a relatively small sample size, which may limit generalizability. Second, the pre–post design may be subject to the Hawthorne effect. Third, given the multiple sub-dimensions analyzed in the scales, we acknowledge the potential for Type I error due to the multiple comparisons problem. However, the primary outcomes (total scores of the scales) demonstrated high statistical significance (*P* < 0.01), suggesting robust findings. Finally, this study focused primarily on process indicators and did not directly assess patient clinical outcomes. Future research should consider a longitudinal design with a follow-up period (e.g., 6 months post-implementation) to evaluate the sustainability of the ISBAR model's effects and to determine if the improvements in critical thinking and satisfaction are maintained over time.

## Conclusion

The ISBAR bedside handover communication model effectively standardized handover processes in a pediatric hematology–oncology department, improved handover quality, reduced nursing handover problems, enhanced nurses' critical thinking disposition, and increased satisfaction among nurses and patients' family members. The model demonstrates substantial clinical value and merits wider application in pediatric specialty settings.

## Data Availability

The raw data supporting the conclusions of this article will be made available by the authors, without undue reservation.
